# Provider’s Perception of Parental Anxiety in the Pediatric Intensive Care Unit

**DOI:** 10.7759/cureus.28589

**Published:** 2022-08-30

**Authors:** Salim Aljabari, Esma Birisci, Faith Kummerfeld

**Affiliations:** 1 Pediatrics, University of Arkansas for Medical Sciences, Little Rock, USA; 2 Statistician, Bursa Uludağ University, Istanbul, TUR; 3 Pediatrics, University of Missouri, Columbia, USA

**Keywords:** family support, family engagement, pediatric intensive care unit, parental anxiety, parental stress

## Abstract

Parents of critically ill children in the Pediatric Intensive Care Unit (PICU) commonly experience new or worsening anxiety, which can lead to long-term sequelae in the form of post-traumatic stress disorder (PTSD). To investigate how well the PICU providers recognize and assess parental anxiety, we assessed the acute and baseline anxiety level of 30 parents in the PICU with the State-Trait Anxiety Inventory (STAI) and compared the results with the PICU physician's and nurses' assessments. All but four parents experienced higher acute anxiety scores compared to baseline, with a 34% increase in the number of parents with moderate and high anxiety scores. All PICU providers performed poorly in recognizing and assessing parental anxiety, with a tendency to underestimate the level of anxiety. Proper screening tools and strategies are essential to recognize and help parents in distress and potentially prevent long-term psychological sequelae.

## Introduction

Parents of critically ill children in the Pediatric Intensive Care unit (PICU) experience several psychological, emotional, physical, financial, and social stressors, all of which commonly lead to new onset or worsening anxiety [1-4]. Many stressors stem from their child's condition, while many others stem from the significant disruption to their daily life and concern about their many responsibilities [1,4].

 Parents fear what could happen to their children. They feel overwhelmed by the complexity of their child's illness and all the decisions they must make. They feel helpless and unable to assume their regular role as parents. They often feel helpless while their beloved, ill-appearing child undergoes painful procedures. They experience sleep deprivation and fatigue from staying in a noisy environment. They abandon their everyday life responsibilities but still have to take care of other family members, work duties, and potential financial responsibilities [2,4,5].

The stress and anxiety parents experience in the PICU can lead to long-term sequelae in the form of post-traumatic stress disorder (PTSD) [5-8]. It has been reported that up 42% of the parents of critically ill children develop PTSD [9-12]. In the United States, more than 230,000 patients are admitted to the PICU annually [13]. This puts hundreds of thousands of parents at risk of experiencing anxiety and developing PTSD every year.

While parental stress and anxiety in the PICU are well reported, and many sources of the stress and anxiety have been identified, we lack studies on how to recognize and adequately assess parental anxiety in the PICU [4,5]. Although all parents of children in the PICU experience acute stress, not all develop anxiety [11,14]. The development of acute anxiety was shown in studies to be unrelated to how sick their child is [11].

It is unclear if PICU providers can accurately recognize parents with a high level of anxiety or not without the use of a validated screening tool. In this study, we aim to investigate whether PICU providers' perception of parental anxiety levels is accurate. We hypothesize that, in general, we underestimate parental anxiety levels and those bedside nurses are better at assessing parental anxiety levels.

## Materials and methods

After obtaining study approval from the University of Missouri Institutional Review Board, the primary investigator (PI) and the PICU social worker would screen the PICU lists, when possible, for potential candidates during the July/1/2020 to June/30/2021. As the study was not funded, the screening and recruitment varied depending on the availability of the PI and social worker. We included parents of critically ill children under 18 during their second or third calendar day in the PICU. Parents were not approached on the first day of PICU admission as they most likely were overwhelmed, tired, and busy arranging the hospital stay. We did not approach parents beyond the third day in the PICU to avoid bias created by the duration of the PICU stay.

We excluded children older than 17 years, planned PICU admission like post-operative admissions, planned transfer to a lower level of care on the day of screening, and instances when non-biological parents were the child's legal guardians or when the state had custody over a child's care. We did not screen for potential candidates when the hospital visitation policy was restricted to only one person due to the pandemic, as the absence of a supporting partner, relative, or friend can potentially increase the stress. Additionally, we excluded parents of children with COVID-19-related illnesses.

To assess the parents' anxiety level, we used the State-Trait Anxiety Inventory (STAI), a well-validated tool for assessing adult anxiety. The inventory has two parts, and each part consists of 20 questions with scores ranging from 20-80. The higher the score, the higher the anxiety level. The Trait-form (T-form) assesses the baseline anxiety level, while the State-form (S-form) assesses the current anxiety level. The STAI enabled us to assess the overall anxiety level of the parents, as well as the change in the anxiety level caused by the PICU admission [15-17].

The PICU social worker approached the parents who met the inclusion criteria. Those who agreed to participate and signed the consent form were then left with a copy of the strait-trait anxiety questionnaires. Once informed consent was obtained from the parent of a child, the social worker asked the attending physician, resident physician, and the bedside nurse of the day whether they thought the anxiety level of the parent was low, moderate, or high based on their interaction with the parent that morning. All physicians and nurses who participated in the study signed a consent form. If the parent did not complete and return the form by the following day, they were excluded from the study.

When only one parent was at the bedside, that parent was asked if s/he was willing to participate. If both parents were at the bedside, the social worker explained the study to them and let them decide who would complete the survey. We recorded whether the mother or father completed the forms.

The primary investigator performed a manual chart review of the children of the participating parents to record the patient's demographics and illness specifics, including primary diagnosis, type of illness, and the child's condition. Additionally, he recorded the type of insurance, and their past medical history was also accounted for.

Standard descriptive statistics were used to describe the patients' characteristics. We used the ANOVA test to compare the variables. Results are presented as mean (standard deviation), and a p-value <0.05 was considered significant. IBM SSPSS 23.0 (SPSS Inc, Chicago, IL) was used for the analysis. 

## Results

We enrolled 30 parents of critically ill children in the study. Three of the 30 participants were fathers, and the rest were mothers. Three mothers were single, two fathers were single, and the rest were married. The parent's demographics and characteristics are highlighted in Table 1.

**Table 1:** Highlight the parents’ demographics.

Category		N (%)
Parent	Mother	27 (90)
	Father	3 (10)
Marital Status	Married	25 (83)
	Single	5 (17)
Race/Ethnicity	Caucasian – non-Hispanic	26 (87)
	African American – non-Hispanic	3 (10)
	Caucasian – Hispanic	1 (3)
Two-Parents household	Yes	26 (87)
	No	4 (13)
Insurance	Private	15 (50)
	Medicaid	11(37)
	Self-Pay	4 (13)

Most children (76%) were younger than three years of age, and 47% were females. Respiratory illness was the most common indication for admission, and four patients (13%) had a dilemma regarding their diagnosis upon the survey's completion. One-third of the patients have been hospitalized before, and 20% have been admitted to the PICU. Forty-three percent of the patients were on mechanical ventilation, and 10% were on vasoactive agents when the survey was completed. None of the patients were on renal replacement therapy, required intracranial pressure monitoring, or were expected to expire when completing the survey. The patients' demographics and characteristics are highlighted in table 2.

**Table 2:** highlight the patient's demographics. CNS: central Nervous System; PICU: Pediatric Intensive Care Unit; RA: Room Air; LFNC: Low Flow Nasal Cannula; NIPPV: Non-Invasive Positive Pressure Ventilation.

Category		N (%)
Age	< 1 year	13
	> 1 year	17
Gender	Female	14
	Male	16
Primary Illness:	Respiratory	23
	Cardiovascular/ shock	3
	CNS	2
	Surgical	2
Condition	Improving	7
	Stable	12
	Worsening	11
Race/Ethnicity	Caucasian – non-Hispanic	26
	African American – non-Hispanic	3
	Caucasian – Hispanic	1
Previous hospitalization	Yes	10
	No	20
Previous PICU stay	Yes	6
	No	24
Chronic medical problems	Yes	11
	No	19
Dilemma regarding diagnosis	Yes	4
	No	26
Respiratory support	RA	2
	LFNC	0
	HFNC	12
	NIPPV	3
	Mechanical Ventilator	13
Vasoactive agents	Yes	3
	No	27

On the trait score, two third of the parents had low scores, one-third had moderate scores, and none had high scores. On the state score, 14 parents (47%) had moderate scores, and five (17%) had high scores. All but four parents had an increase in their anxiety score, with 16 parents (53%) having an increase of 10 points or more. The average (SD) increase in the score was 11.2 (8).

The increase in the anxiety scores significantly correlated with the child's age and history of the previous hospitalization. Parents with children younger than one year of age were more likely to experience a higher level of anxiety (15.4 (7) vs. 8.8 (8), P = 0.038). History of the previous hospitalization was associated with a significantly lower score change (6.5 (5) vs. 13.6 (9), P = 0.029). Last PICU admission was not associated with a lower score change (7 (5) vs. 12.3 (9), P = 0.17). As highlighted in Table 3, all the other patients' and parents' variables did not correlate with the change in the anxiety score.

**Table 3:** association between patient’s and parent’s variables and the change in the anxiety score from baseline. PICU: Pediatric Intensive Care Unit; RA: Room Air; LFNC: Low Flow Nasal Cannula; NIPPV: Non-Invasive Positive Pressure Ventilation.

Variable		Change in anxiety score Mean(SD)	P value
Age	< 1 year	15.3 (7)	0.03
	> 1 year	8.8 (8)	
Gender	Female	9.7 (8)	0.35
	Male	12.6 (9)	
Condition	Improving	9.1 (8.6)	0.39
	Stable	9.8 (8)	
	Worsening	14 (9)	
Previous hospitalization	Yes	6.5 (5)	0.02
	No	13.6 (9)	
Previous PICU stay	Yes	7 (5)	0.17
	No	12.2(9)	
Chronic medical problems	Yes	10 (8)	0.56
	No	11.9 (9)	
Dilemma regarding diagnosis	Yes	9.5 (7)	0.67
	No	11.5 (9)	
Respiratory support	RA	9 (11)	0.85
	HFNC	10 (9)	
	CPAP/BiPAP	10.6 (1)	
	Mechanical Ventilator	12.8 (8)	
Marital Status	Married	11.3(9)	0.91
	Single	10.8(8)	
Two-Parents household	Yes	11.2(9)	0.96
	No	11(4)	
Insurance	Private	10.8(9)	0.96
	Medicaid	11.5(6)	
	Self-Pay	11.8(13)	

Attending physicians' perception of the parent's anxiety level was accurate 57% of the time, the bedside nurse's perception was accurate 50%, and the resident physician's perception was accurate 37% of the time. Attending physicians and nurses underestimated the anxiety severity 27% of the time, and resident physicians only underestimated the severity 17% of the time and overestimated the anxiety severity 45% of the time. Among the five parents with high state-anxiety scores, the attending physicians and bedside nurses underestimated the anxiety level in four out of the five parents (80%).

## Discussion

As we care for critically ill children, it is essential to keep in mind the well-being of the parents and family. Recognition of parental stress and anxiety allows for early intervention and more focused family support. Liaw et al., in a quality improvement initiative, screened the parents of critically ill children for stress and provided focused support [18]. They reported better family satisfaction and lower parental distress [18]. Our study reaffirms the magnitude of stress and subsequent anxiety that parents of critically ill children experience; and demonstrates poor recognition and assessment of parental anxiety when the provider's perception, rather than a validated screening tool, is used.

The long-term psychological sequelae on parents of PICU patients is somewhat of a mental health crisis. Logan et al. identified 95,070 parents whose children were admitted to the PICU between 2006 to 2013 [7]. Within six months of the PICU admission, 9.5 % of them had developed a new mental health diagnosis, representing a 110% increase compared to before the PICU admission [7]. When we put this in perspective, with more than 230,000 children admitted to the PICU annually in the United States [13], we expect more than 20,000 parents annually to have a new mental illness diagnosis due to their PICU experience. 

Using the State-Trait Anxiety inventory, we could assess the baseline anxiety level and the change in the score from the PICU admission [15,16]. Our analysis focused on the change from baseline score to avoid bias created by baseline anxiety. We showed a 17% increase in parents with moderate anxiety scores and parents with high anxiety scores. We also demonstrated that more than half of the parents scored 10 points or higher than baseline. The high rate of anxiety among parents in the PICU in our study is very comparable to previous studies [19].

Both physicians and nurses failed to assess the anxiety level of the parents accurately. They especially performed poorly in the subcategory of parents with very high anxiety scores, underestimating their anxiety 80% of the time. The subgroup with very high anxiety scores is the most vulnerable and at risk for long-term sequelae. Previous adult and neonatal studies reported similar findings [20-22]. Nurses in adult ICUs poorly assessed stress and anxiety in their patients [20,21]. While we can assume that with experience, PICU providers can perform better as the attending physicians performed better than their trainees, the improvement did not reach a satisfactory level.

In the PICU, nurses spend long hours with patients and interact with their parents more frequently than physicians. Besides delivering most of the ICU care and closely monitoring the patients, they are essential in providing the parents with updates and emotional support when needed [23]. With that, we hypothesized that bedside nurses are more likely to recognize parental stress and anxiety than physicians. The fact that the nurses missed the mark in almost half of the patients and were not better than physicians at recognizing parents with a very high-stress level is an important signal that perception of anxiety based on usual interaction is a poor approach. This finding calls for developing and employing a standard screening tool for parental anxiety in the PICU.

PICU providers, especially attending physicians and nurses, were likelier to underestimate than overestimate parents' anxiety. PICU nurses and attending physicians underestimated the anxiety level in 27% of the cases. Although resident physicians overestimated much more than underestimated, all the providers, including resident physicians, underestimated the parents with very high scores. The risk of missing parents with high anxiety levels is far more troubling than any potential harm of overestimating their anxiety level.

Our findings support previous studies in that the severity of the child's illness, the need for invasive interventions, and the overall prognosis does not correlate with parental anxiety [6]. Assuming that a parent is not distressed and anxious simply because their child is not critically ill and has an excellent prognosis is a natural behavior from an ICU practitioner. Ballufi et al. reported high rates of acute stress disorder among parents in the PICU, which was associated with high rates of PTSD in the long term [11], and neither the acute stress nor the PTSD correlated with the PRISM III score [11]. As the child's condition is one of many sources of stress, and the parent's perception of their child's condition might not correlate with their actual condition, this assumption is misleading. Additionally, several studies reported that all parents in the PICU experience stress [14], but not all of them develop anxiety [11]; we should screen for parental anxiety rather than stress.

The parent's socioeconomic status, including insurance type, employment status, and marital status, did not Corollate with the change in their anxiety. While it is possible that our study was underpowered to detect the effect of the psychosocial status on acute parental anxiety, it is also possible that the reason some parents develop acute anxiety and others do not is very complex. A more detailed assessment of personal and social factors is needed to identify risk factors.

We believe that recognizing anxiety among parents in the PICU is the first step in providing attentive care to prevent long-term psychological sequelae and mental illnesses. The PICU team should investigate the source of stress and anxiety and intervene accordingly. Interventions can be psychosocial support, a better explanation of the illness and prognosis, more involvement in the care of their child, or even financial/insurance aid. Previous studies reported several modifiable sources of parental stress and anxiety, such as the loss of the parental role, their perception of the child's condition and prognosis, the noises in the PICU, the lack of privacy, and financial and life responsibilities outside the PICU [1,2,5].

Our study has several limitations. First, the small sample size did not detect the risk factors associated with parental anxiety. However, despite that, our study showed a higher incidence of increased anxiety scores and poor recognition by the providers, which is the primary goal of this study. Second, the PICU at the University of Missouri Children's Hospital is a small to medium size unit, does not offer many advanced interventions, including extracorporeal membrane oxygenation or organ transplantation, and is not a trauma center. Our PICU's patient population is lower acuity than larger academic centers. Despite that, our anxiety rates are similar to those reported from bigger centers19. Moreover, that is again evidence that parental anxiety in the PICU is high regardless of how sick a child is. Furthermore, finally, due to the small sample size of PICU nurses, we did not collect data on their years of experience, which could affect how accurately they estimate parental anxiety.

## Conclusions

Most parents of critically ill children experience acute anxiety. Parental anxiety in the PICU did not correlate with their child's illness severity, prognosis, previous PICU admission, or psychosocial background. Physicians and nurses failed to assess parental anxiety level half of the time accurately. Attending physicians and nurses were more likely to underestimate the anxiety level than to overestimate it, with all providers underestimating the anxiety level in the subgroup of parents with very high anxiety scores 80% of the time. Screening parents in the PICU for anxiety via a validated tool and early intervention to potentially prevent long-term sequelae is essential.

## References

[1] Hagstrom S (2017). Family stress in pediatric critical care. J Pediatr Nurs.

[2] Gallegos CM (2011). An examination of parental stress and coping in the pediatric intensive care unit (PICU).

[3] Day A, Haj-Bakri S, Lubchansky S, Mehta S (2013). Sleep, anxiety and fatigue in family members of patients admitted to the intensive care unit: a questionnaire study. Crit Care.

[4] Christian BJ (2020). Translational research - parental stress associated with hospitalization of children with critical life-threatening conditions and the long-term impact. J Pediatr Nurs.

[5] Abela KM, Wardell D, Rozmus C, LoBiondo-Wood G (2020). Impact of pediatric critical illness and injury on families: an updated systematic review. J Pediatr Nurs.

[6] Fumis RR, Ranzani OT, Martins PS, Schettino G (2015). Emotional disorders in pairs of patients and their family members during and after ICU stay. PLoS One.

[7] Logan GE, Sahrmann JM, Gu H, Hartman ME (2020). Parental mental health care after their child’s pediatric intensive care hospitalization. Pediatr Crit Care Med.

[8] Van Horn E, Tesh A (2000). The effect of critical care hospitalization on family members: stress and responses. Dimens Crit Care Nurs.

[9] Colville G, Pierce C (2012). Patterns of post-traumatic stress symptoms in families after paediatric intensive care. Intensive Care Med.

[10] Bronner MB, Kayser AM, Knoester H, Bos AP, Last BF, Grootenhuis MA (2009). A pilot study on peritraumatic dissociation and coping styles as risk factors for posttraumatic stress, anxiety and depression in parents after their child's unexpected admission to a pediatric intensive care unit. Child Adolesc Psychiatry Ment Health.

[11] Balluffi A, Kassam-Adams N, Kazak A, Tucker M, Dominguez T, Helfaer M (2004). Traumatic stress in parents of children admitted to the pediatric intensive care unit. Pediatr Crit Care Med.

[12] Rees G, Gledhill J, Garralda ME, Nadel S (2004). Psychiatric outcome following paediatric intensive care unit (PICU) admission: a cohort study. Intensive Care Med.

[13] Kahn JM, Le T, Angus DC (2015). The epidemiology of chronic critical illness in the United States*. Crit Care Med.

[14] Board R, Ryan-Wenger N (2003). Stressors and stress symptoms of mothers with children in the PICU. J Pediatr Nurs.

[15] Spielberger CD (1983). State-Trait Anxiety Inventory for Adults. American Psychological Association.

[16] Spielberger Spielberger, C. D. (2022). The State-Trait Anxiety Inventory. https://www.mindgarden.com/150-test-anxiety-inventory#horizontalTab3.

[17] Ugalde A, Krishnasamy M, Schofield P (2014). The relationship between self-efficacy and anxiety and general distress in caregivers of people with advanced cancer. J Palliat Med.

[18] Liaw KR, Cho J, Devins L, Daly J, Sklenar D, Al-Qaqaa Y (2019). Co-designed PICU family stress screening and response system to improve experience, quality, and safety. Pediatr Qual Saf.

[19] Needle JS, O'Riordan M, Smith PG (2009). Parental anxiety and medical comprehension within 24 hrs of a child's admission to the pediatric intensive care unit*. Pediatr Crit Care Med.

[20] Pang PS, Suen LK (2008). Stressors in the ICU: a comparison of patients' and nurses' perceptions. J Clin Nurs.

[21] Novaes MA, Knobel E, Bork AM, Pavão OF, Nogueira-Martins LA, Ferraz MB (1999). Stressors in ICU: perception of the patient, relatives and health care team. Intensive Care Med.

[22] Satnarine T, Ratna P, Sarker A (2022). The relationship between infant prematurity and parental anxiety: a systematic review. JMHS.

[23] Gaeeni M, Farahani MA, Seyedfatemi N, Mohammadi N (2014). Informational support to family members of intensive care unit patients: the perspectives of families and nurses. Glob J Health Sci.

